# GmZPR3d Interacts with GmHD-ZIP III Proteins and Regulates Soybean Root and Nodule Vascular Development

**DOI:** 10.3390/ijms20040827

**Published:** 2019-02-14

**Authors:** Suresh Damodaran, Amélie Dubois, Juan Xie, Qin Ma, Valérie Hindié, Senthil Subramanian

**Affiliations:** 1 Department of Agronomy, Horticulture and Plant Science, South Dakota State University, Brookings, SD 57007, USA; sureshd@wustl.edu (S.D.); amelie2807@hotmail.com (A.D.); qin.ma@sdstate.edu (Q.M.); 2 Department of Biology, Washington Univeristy in St. Louis, St. Louis, MO 63130, USA; 3 Ecole Nationale Supérieure Agronomique, Avenue de l’Agrobiopole, BP32607 Auzeville-Tolosane, France; 4 Department of Mathematics and Statistics, South Dakota State University, Brookings, SD 57007, USA; Juan.xie@sdstate.edu; 5 Hybrigenics Services, 3-5 Impasse Reille, 75014 Paris, France; hindie.valerie@gmail.com; 6 Department of Biology and Microbiology, South Dakota State University, Brookings, SD 57007, USA

**Keywords:** ZPR, HD-ZIP III, soybean, nodule, yeast two-hybrid, vasculature development

## Abstract

Fabaceans produce two major classes of symbiotic nodules: the indeterminate type characterized by a persistent meristem, and the determinate type that lacks a persistent meristem. The class III homeodomain leucine zipper (HD-ZIP III) transcription factor family influence development of multiple lateral organs and meristem maintenance, but their role in determinate nodule development is not known. HD-ZIP III protein activity is post-translationally regulated by members of the small leucine zipper protein (ZPR) family in arabidopsis. We characterized the ZPR gene family in soybean and evaluated their ability to interact with two key members of GmHD-ZIP III family through yeast two-hybrid assays. GmZPR3d displayed the strongest interaction with GmHD-ZIP III-2 among the different pairs evaluated. GmHD-ZIP III-1, -2, and GmZPR3d showed overlapping expression patterns in the root stele and in nodule parenchyma tissues. Over-expression of GmZPR3d resulted in ectopic root secondary xylem formation, and enhanced expression of vessel-specific master switch genes in soybean. The nodules in ZPR3d over-expressing roots were larger in size, had a relatively larger central zone and displayed increased nodule vascular branching. The results from this study point to a key role for GmZPR3d in soybean root and nodule development.

## 1. Introduction

Class III homeodomain leucine zipper proteins (HD-ZIP IIIs) are plant specific transcription factors and are key players in multiple developmental processes beginning from embryo development to plant maturity in Arabidopsis and other plant species [1,2,3]. HD-ZIP IIIs are multi-domain proteins and are distinguished from other classes of homeodomain and leucine zipper families based on the presence of both these domains (as reviewed by [4,5]). The homeobox domain is similar to that found in drosophila and other genera, but the presence of a leucine zipper domain adjacent is unique to plants; and the leucine zipper domain is responsible for dimerization and enabling the homeodomains to bind to the DNA fragment to perform its role as a transcriptional factor. Other domains present in HD-ZIP III proteins include a START domain, a SAD domain and a MEKHLA domain [6].

There are five HD-ZIP III genes in Arabidopsis: *ATHB14*/*PHABULOSA (PHB)*, *PHAVOLUTA (PHV)*, *REVOLUTA (REV)*, *ATHB8* and *ATHB15/CORONA*. Several loss- or and gain-of-function mutants of these genes have been shown to affect shoot apical meristem (SAM) and lateral organ polarity [7]. Loss of function single mutants of HD-ZIP III proteins do not show any obvious phenotypic defects, but multi-order mutants such as *phb-6/phv-5/rev-9* lack a shoot apical meristem and have a single abaxialized cotyledon indicating overlapping/redundant functions of HD-ZIP III proteins [3]. Similarly, while loss of function mutations in *ATHB8* do not show any defects in vascular development, overexpression of this gene results in increased xylem formation and perturbed radial arrangement of xylem and phloem cells due to ectopic differentiation of procambial cells [8]. Role of *ATHB15* in root development was discovered with the gain of function allele *icu4-1*. This mutant has a disorganized root meristem with excess cells around the quiescent center (QC) and an increased number of lateral roots. Similarly, the gain of function mutant *rev-10d* also has increased number of lateral roots [1]. In agreement with this, the triple loss of function mutant *phb-6/phv-5/rev-9* shows reduced number of lateral roots [1]. All these studies clearly indicated a role for HD-ZIP IIIs in vascular development and lateral organ formation.

Little is known about the role of HD-ZIP III proteins in symbiotic nodule formation in legumes. Symbiotic nodules are specialized root lateral organs formed as a result of mutually beneficial interactions between leguminous plants and nitrogen fixing rhizobia present in the soil. Rhizobia convert atmospheric nitrogen to plant usable forms and receive carbon in the form of organic acids from the plant [9]. There are two major types of nodules among leguminous plants: determinate and indeterminate (as reviewed by [10]). Though both classes of nodules support nitrogen fixation, their structure and some developmental programs are different. Determinate nodules typically arise from outer cortical cells of root, do not possess a persistent meristem, and appear spherical in shape upon maturity; examples include soybean (*Glycine max*) and *Lotus japonicus*. Most indeterminate nodules arise from inner cortical cells of the root with a few exceptions, and possess a persistent meristem which gives rise to an oblong mature nodule; examples include *Medicago truncatula* and white clover (*Trifolium repens*). In both the determinate and indeterminate nodules, vascular bundles are observed in the periphery, and are crucial for the exchange or transport of nutrients especially nitrogenous compounds and carbon [11,12]. The soybean root nodule is known to anatomically possess a system of several looping and branching vascular bundles to cover the central infection zone along the nodule parenchyma. The function of a number of signaling components and transcription factors are conserved between the two types of nodules. For example, cytokinin signaling is necessary for the initiation of both types of nodules [13,14] and the roles of nod factor receptors and downstream signaling elements are conserved between the two types of nodules [15,16]. However, some signaling elements appear to be specific to each nodule type. For example, while NAC transcription factors targeted by microRNA164 (miR164) appear to play a key role in the development of indeterminate nodules, they do not appear to be important for determinate nodule development [17]. In *M. truncatula* down-regulation of HD-ZIP III gene expression through overexpression of microRNA166 (miR166) reduced the numbers of nodules and lateral roots, and induced ectopic root xylem development [18]. This suggested that HD-ZIP III activity might be crucial for the development of indeterminate nodules. However, their role in determinate nodule development is not known.

HD-ZIP III protein activity is regulated at the post-translational level by members of a small leucine zipper family (ZPR). Two of the family members, ZPR1 and ZPR3, were up-regulated in Arabidopsis plants over-expressing REV [19]. In situ hybridization experiments revealed an overlapping expression pattern of ZPRs with REV in SAM and leaves. Since dimerization among HD-ZIP III proteins is essential for their transcriptional activity, interaction of ZPRs with HD-ZIP III was evaluated. Interestingly all four members of the ZPR family showed positive interaction with REV or PHB resulting in the formation of heterodimers. Over-expression of ZPR3 protein caused downward curling of leaves and disorganized SAM reminiscent of plants with reduced HD-ZIP III. This was similar to the phenotype observed in plants with reduced HD-ZIP III activity, e.g., those that over-express microRNA166. This suggested that ZPR proteins might reduce HD-ZIP III transcriptional activity through heterodimerization. A feedback loop mechanism was hypothesized where HD-ZIP III proteins induce the expression of *ZPR* genes and in turn ZPR proteins inhibit HD-ZIP III activity by forming heterodimers [6,19,20].

To evaluate if HD-ZIP III activity is crucial for determinate nodule development, we identified HD-ZIP III and ZPR family members in soybean, and evaluated the interaction of soybean ZPR proteins with two selected soybean HD-ZIP III proteins. Results from yeast two-hybrid assays identified specific ZPR members with distinct affinities to the HD-ZIP IIIs tested. Microscopic imaging of promoter Tdtomato fusions revealed overlapping expression patterns of the positively interacting ZPR and the two HD-ZIP III genes in roots and nodules. Overexpression of GmZPR3d altered root stele development, and nodule structure, but not nodule numbers. Our results suggest that HD-ZIP III activity is crucial for proper nodule development in soybean. Interaction of GmZPR3d with a wide range of proteins in a genome-wide yeast two-hybrid screen suggested that its regulatory role might extend beyond HD-ZIP III proteins.

## 2. Results

### 2.1. Identification of Soybean HD-ZIP III and ZPR Genes in Soybean

**Figure 1:**
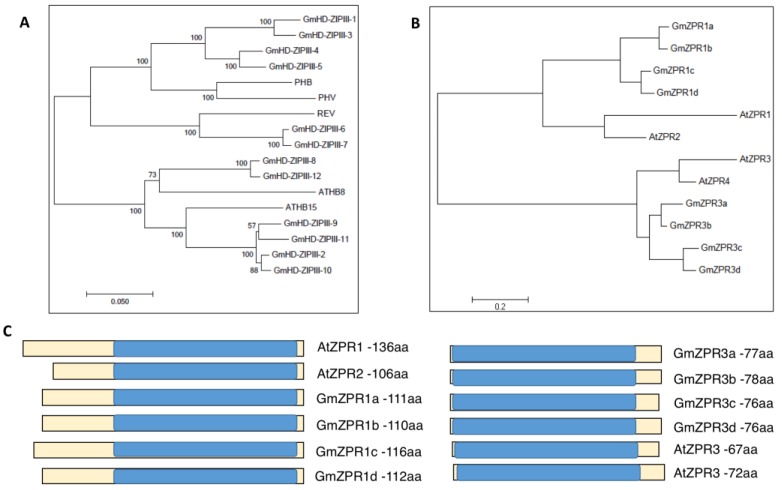
Identification of GmHD-ZIPIII and GmZPR genes in soybean. (**A**) Phylogenetic tree based on peptide sequence showing four major clades of soybean GmHD-ZIP III proteins, and the closest potential Arabidopsis ortholog using Neighborhood joining method. (**B**) Phylogenetic Tree based on peptide sequencing showing two major clades of soybean GmZPR protein and their closest potential Arabidopsis ortholog using Neighborhood joining method. (**C**) Protein domains of GmZPR peptides aligned with those of AtZPRs with the leucine zipper domain highlighted in blue. The length of each peptide is also indicated.

To identify HD-ZIP III genes in *G. max*, a BLASTp search was performed against the *G. max* genome sequence (version 1.89; https://phytozome.jgi.doe.gov), using arabidopsis HD-ZIP III sequences as query. The search returned twelve putative *G. max* HD-ZIP III genes which were named GmHD-ZIP III-1 to -12. The gene IDs and expression patterns from soybean transcriptomic atlas of these genes are presented in Table S1A. A phylogenetic tree was constructed using the peptide sequences of soybean and arabidopsis HD-ZIP III genes to determine potential evolutionary relationships. The phylogenetic tree displayed four distinct branches consisting of (i) PHB and PHV, (ii) REV, (iii) ATHB8 and (iv) ATHB15 (Figure 1A). The twelve GmHD-ZIP IIIs fell into these four branches. ATHB15 was distinct in that it had four potential soybean orthologs compared to just two potential orthologs for the other arabidopsis HD-ZIP III proteins (Figure 1A). This suggested that ATHB15 sub-family might have diversified further with potential additional biological roles in soybean.

To identify soybean ZPRs, peptide BLAST searches were performed as above using arabidopsis ZPR sequences as query. The results identified four *G. max* genes homologous to AtZPR1 and 2; these had a leucine zipper motif at the C terminal region and an unknown peptide domain at the N terminal region (Figure 1B,C and Figure S1). Similarly, four other soybean genes homologous to AtZPR3 and 4 were identified. These had only the leucine zipper motif and lacked the N terminal domain found in the ZPR1 and 2 classes of proteins (Figure 1B,C and Figure S1). The leucine zipper domain was examined manually for the presence of leucine residues in heptads positions since this is crucial for dimerization. All eight GmZPR proteins had a leucine/isoleucine residue at position “d” of the heptad series suggesting that they are capable of forming the hydrophobic core promoting homo/heterodimerization with leucine zipper proteins [21,22] (Figure S1B). In other words, the identified ZPRs are not only similar in peptide sequence, but also possess the appropriate residues at the expected positions for functionality. GmZPR genes belonging to the same class showed high similarity in peptide sequences between each other in the sequence alignment (Figure S1A,B). For example, GmZPR3a had identical amino acid sequence to that of GmZPR3b in the heptad repeats of the potential leucine-zipper domain, and showed an overall 91% peptide sequence identity with the latter gene (Figure S1B,C). The Gene IDs and expression patterns from soybean transcriptomic atlas of these genes are presented in Table S1B.

### 2.2. GmZPR3d Displayed Strong Interaction Capacity with GmHD-ZIP III Proteins

In results from phylogenetic analysis, GmHD-ZIP III -1 clustered together with GmHD-ZIP IIIs 3, 4 and 5, and presented close similarity to the Arabidopsis HD-ZIP III proteins PHB and PHV. GmHD-ZIP III-2 clustered together with GmHD-ZIP IIIs -9, -10, and -11, and showed close similarity to ATHB15. Given the roles of Arabidopsis PHB, PHV, and ATHB15 in lateral organ and meristem function, we evaluated the interaction of GmZPR proteins with GmHD-ZIP III-1 and -2 using yeast two-hybrid assays. All the eight *GmZPR* genes were cloned in frame with GAL4 DNA-binding domain and *HD-ZIP III* genes with GAL4 activation domain and subject to yeast two-hybrid assays, along with positive (pGBKT7-T & pGADT7-53) and negative control (pGBKT7-T & pGADT7-Lam) protein pairs provided by the manufacturer of the yeast two-hybrid assay kit (Takara Bio USA, Mountain View, CA, USA). Both HD-ZIP III proteins displayed autoactivation when fused with GAL4 DNA-binding domain, but not with GAL4 activation domain. None of the ZPR proteins displayed autoactivation in either fusion (Figure S2). Mated yeast cells harboring different combinations of vectors were plated at multiple dilutions on different selection media and growth characteristics observed once a day for 15 days to obtain preliminary quantitative estimates on the interaction.

**Figure 2:**
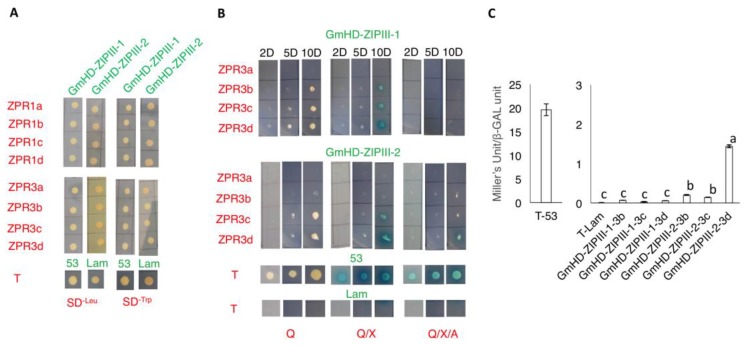
Interaction of GmZPRs with GmHD-ZIP III-1 and -2 in yeast-2-hybrid assays. (**A**) Colonies of mated yeast cells containing GmHD-ZIPIII-1 or -2 and one of the GmZPRs grown on SD-leu and SD-Trp. Yeast cells containing T-53 and T-Lam were used as positive and negative controls respectively. (**B**) Colonies of mated yeast cells containing GmHD-ZIP III-1 or -2 and one of the GmZPR3 family members grown on different assay plates imaged at 2, 5, and 10 days after plating. Q refers to minimal media without Leu, Trp, Ade and His; X- with X-α-Gal; and A-with Aureobasidin. Yeast cells containing T-53 and T-Lam were used as positive and negative controls respectively. (**C**) β-GAL activity of mated yeast cells containing GmHD-ZIPIII-1 or -2 and one of GmZPR3b, 3c, or 3d assayed using a spectrophotometric assay. Yeast cells containing T-53 and T-Lam were used as positive and negative controls respectively. Data shown are average Miller’s units normalized to yeast cell concentration from three replicate assays. Error bars indicate SE (*n* = 3). Samples marked with different letters are significantly different from each other based on One-way ANOVA and Tukey HSD test.

Positive control yeast clones (T::53) produced colonies as early as 2 days after plating. However, negative control clones (T::Lam) did not produce any colonies even after 15 days (Figure 2A,B). Positive control cells grew at equal rates in SD/-Leu/-Trp (selects for the presence of plasmids), SD/QDO (selects for activation of auxotrophic markers through positive interaction), SD/QDO/ X-α-Gal (selects for activation of auxotrophic and chromogenic marker through positive interaction), and SD/QDO/X-α-Gal/AbA (selects for activation of auxotrophic, chromogenic and antibiotic resistance markers through positive interaction). This indicated positive interaction between the positive control proteins to activate transcription of all the four different marker genes. Yeast cells bearing all GmZPR::HD-ZIP III pairs produced colonies on SD/-Leu/-Trp plates indicating that they were viable (Figure 2A). On interaction assay plates, only yeast cells bearing GmZPR3b, c, or d and either of the GmHD-ZIP III proteins showed growth indicating that only these pairs had positive protein-protein interaction in the yeast two-hybrid assays.

Colonies from yeast cells expressing GmHD-ZIP III-1 and any of GmZPR3b, c or d started to appear at 2 days after plating on SD/-Leu/-Trp and SD/QDO, and SD/QDO/X-α-Gal plates, similar to the positive control pair (Figure 2B). However, no colonies were seen on SD/QDO/X-α-Gal/AbA plates even at 10 days post plating. At this time point, the colonies were clearly visible and there was blue coloration in SD/QDO/X-α-Gal plates (Figure 2B). On the other hand, yeast cells expressing GmHD-ZIP III-2 and any of GmZPR3b, 3c or 3d produced clearly visible colonies only 5 days post plating, but they displayed growth on SD/QDO/X-α-Gal/AbA at both 5- and 10-days post plating (Figure 2B). The ability to activate all four different marker genes varied between different test proteins as shown by colony growth on AbA suggesting that the strength of interaction might vary between the different pairs evaluated. GmHD-ZIP III-1 or -2 failed to show interaction with any of the GmZPR1 class of proteins (data not shown) [23] or GmZPR3a (Figure 2B) as indicated by absence of yeast growth in any of the assay plates even after 15 days.

A quantitative β-gal assay was performed in addition to the colony growth assays above as an indicator of the strength of interactions between GmZPR3b, 3c and 3d with GmHD-ZIP III -1 and -2. Yeast cells expressing positive control plasmid pairs converted the substrate into product (yellow coloration) within 20–30 min and the intensity developed as the time lapsed but there was not any change in color for the test samples at this time point. Almost 20 h after substrate addition, visible yellow colored product started to appear in test samples. The optical density (OD_420_) of the colored product and blank samples were measured after terminating the reaction to calculate β -gal units (or Miller’s units) of the culture [24]. As expected, T::53 (positive) showed very high β-Gal activity (~14-fold more than any of the experimental pairs; Figure 2C). Among the HD-ZIP III::ZPR pairs, GmHD-ZIP III-2::ZPR3d cells showed highest β-Gal activity, about 7-fold higher than the next group consisting of GmHD-ZIP III-2::ZPR3b and GmHD-ZIP III-2::ZPR3c (Figure 2C). This was in agreement with the ability of cells containing these above interaction pairs to grow on the most stringent assay plate, SD/QDO/X-α-Gal/AbA. Cells containing GmHD-ZIP III-1 and one of ZPR3b, 3c or 3d, showed much lower β-Gal activity. In fact, none of them showed a statistically significantly higher β-Gal activity than the negative control (Figure 2C). We concluded that GmZPR3d displayed the strongest interaction with GmHD-ZIP III-2 among the different pairs evaluated.

### 2.3. GmZPR3d and GmHD-ZIP III-1 and -2 Display Overlapping Expression Patterns in the Root and Nodule Vasculature

ZPR3d showed the strongest interaction with both GmHD-ZIP III-1 and -2 when considering results from both yeast cell growth and β-Gal assays together. Therefore, we evaluated the expression patterns of these three genes in soybean roots and nodules to determine tissue domains with overlapping expression. Presumably, these are tissues where ZPR3d might interact with GmHD-ZIP III-1 and -2 to regulate their activity. Corresponding promoter regions (~1700 bp) upstream of the coding sequences of the above three genes were used to drive the expression of tdTomato in soybean hairy root composite plants. A constitutively expressed pSuperUbi::GFP (green-false color) construct in the same vector was used to identify transgenic roots (Figure S3). To evaluate for non-specific fluorescence, we imaged a control vector that had the GFP cassette, but not the tdTomato cassette (red-false color) using the PI/TRITC filter. No fluorescence was observed in root tips, elongation zone of roots, or emerging nodules at the exposure times used for imaging (Figure S2C,F,I,L). Non-specific fluorescence was observed in mature nodules tissues with longer exposure times (Figure S2O–R).

**Figure 3:**
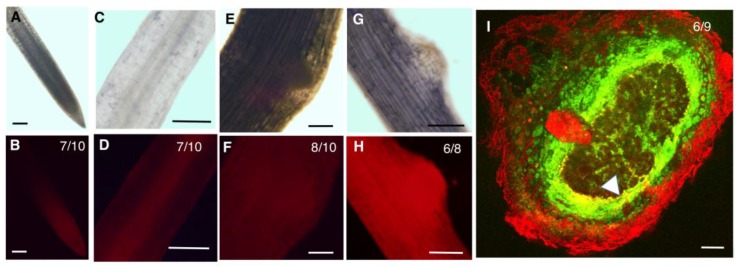
Spatio-temporal expression patterns of GmHD-ZIPIII-1. Expression of GmHD-ZIP III-1p:TdTomato in whole mounts of a soybean root tip (**A**,**B**), mature root region showing the root stele (**C**,**D**), an emerging lateral root (**E**,**F**), and an emerging nodule (**G**,**H**), imaged using a compound fluorescence microscope. **A**, **C**, **E** and **G** are bright field images and **B**, **D**, **F**, and **H** are corresponding fluorescence images obtained using a Propidium Iodide/tetramethylrhodamine isothiocyanate (PI/TRITC) filter. (**I**) A median section of a mature nodule longitudinal to the nodule axis showing expression of GmHD-ZIPIII-1p:TdTomato in the nodule parenchyma imaged using a laser confocal microscope. Tdtomato (red) signal appears yellow due to overlap with a co-expressed constitutive GFP construct. Bright red fluorescence in the nodule and root peripheries and the root stele indicate non-specific fluorescence. See Figure S4 for split channels and color blind-friendly composite image. Scale bars are 100 μm. The number of independent transgenic roots/nodules showing representative patterns out of the total number evaluated are indicated in panels **B**, **D**, **F**, **H** and **I**.

**Figure 4:**
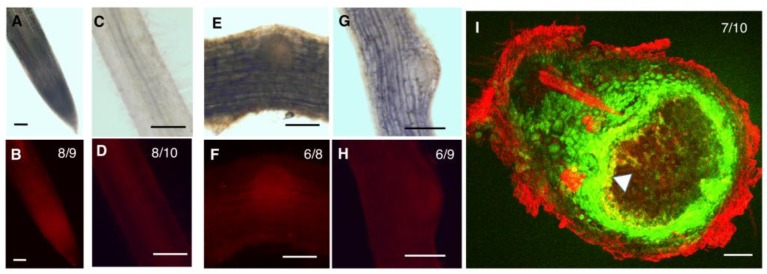
Spatio temporal expression patterns of GmHD-ZIPIII-2. Expression of GmHD-ZIP III-2p:TdTomato in whole mounts of a soybean root tip (**A**,**B**), mature root region showing the root stele (**C,D**), an emerging lateral root (**E**,**F**), and an emerging nodule (**G**,**H**), imaged using a compound fluorescence microscope. **A**, **C**, **E** and **G** are bright field images and **B**, **D**, **F** and **H** are corresponding fluorescence images obtained using a PI/TRITC filter. (**I**) A median section of a mature nodule longitudinal to the nodule axis showing expression of GmHD-ZIPIII-2p:TdTomato in the nodule parenchyma imaged using a laser confocal microscope. Tdtomato (red-false color) signal appears yellow due to overlap with a co-expressed constitutive GFP (green- false color) construct. Bright red fluorescence in the nodule and root peripheries and the root stele indicate non-specific fluorescence. See Figure S4 for split channels and color blind-friendly composite image. Scale bars are 100 μm. The number of independent transgenic roots/nodules showing representative patterns out of the total number evaluated are indicated in panels **B**, **D**, **F**, **H** and **I**.

In the root tip, both GmHD-ZIP III -1 and 2 displayed expression in the meristematic region (Figure 3B and Figure 4B). While GmHD-ZIP III-1 expression was observed in the lateral root cap, GmHD-ZIP III-2 showed little or no expression in the root cap (Figure 3B and Figure 4B). Both GmHD-ZIP III -1 and 2 displayed clearly detectable expression in the root stele (Figure 3D and Figure 4D). While GmHD-ZIP III-1 expression was very low in emerging lateral roots, GmHD-ZIP III-2 had a prominent expression in these tissues (Figure 3F vs. Figure 4F). In contrast, GmHD-ZIP III-1 showed much prominent expression in emerging nodules (7–8 days post rhizobium inoculation) compared to that of GmHD-ZIP III-2 (Figure 3H vs. Figure 4H).

The expression of both GmHD-ZIP III-1 and 2 was readily detectable in mature nodules evaluated at 14 dpi. The large size of mature nodules and the presence of multiple cell layers did not allow us to distinguish or identify tissue-specific expression using whole mount images. Therefore, mature nodules were hand-sectioned and tdTomato fluorescence was imaged using a laser confocal microscope. To distinguish between promoter activity and non-specific fluorescence which is prevalent in mature nodule tissues especially under confocal imaging conditions, we imaged constitutively expressed GFP (driven by the super-Ubiquitin promoter) along with promoter:tdTomato expression. Presence of GFP signal helps distinguish live cells from non-specific fluorescence on the root and nodule periphery and vasculature [25]. GmHD-ZIP III-1p:tdTomato expression (indicated by bright yellow fluorescence in GFP-tdTomato overlays) was prominent in the nodule parenchyma, but not in other nodule tissues (indicated by green fluorescence; Figure 3I). GmHD-ZIP III-2 was also predominantly detected in nodule parenchyma cells, but only in those bordering the central zone (Figure 4I). GmHD-ZIP III-1 expression was much stronger compared to that of GmHD-ZIP III-2 in mature nodules, which was also the case in emerging nodules.

**Figure 5:**
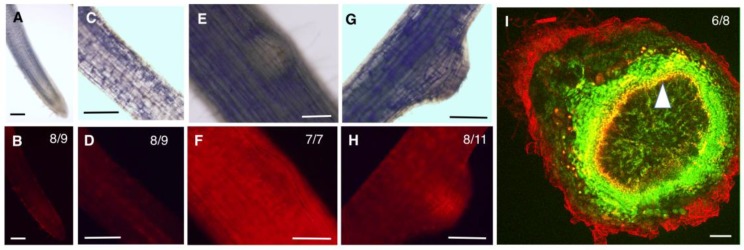
Spatio temporal expression patterns of GmZPR3d. Expression of GmZPR3dp:tdTomato in whole mounts of a soybean root tip (**A**,**B**), mature root region showing the root stele (**C**,**D**), an emerging lateral root (**E**,**F**), and an emerging nodule (**G**,**H**), imaged using a compound fluorescence microscope. **A**, **C**, **E** and **G** are bright field images and **B**, **D**, **F** and **H** are corresponding fluorescence images obtained using a PI/TRITC filter. (**I**) A median section of a mature nodule longitudinal to the nodule axis showing expression of GmZPR3dp:tdTomato in the nodule parenchyma imaged using a laser confocal microscope. Tdtomato (red) signal appears yellow due to overlap with a co-expressed constitutive GFP construct. Bright red fluorescence in the nodule and root peripheries and the root stele indicate non-specific fluorescence. See Figure S4 for split channels and color blind-friendly composite image. Scale bars are 100 μm. The number of independent transgenic roots/nodules showing representative patterns out of the of the total number evaluated are indicated in panels **B**, **D**, **F**, **H** and **I**.

Patterns of GmZPR3dp:tdTomato expression indicated interesting overlaps with those of GmHD-ZIP III-1 and -2. In roots GmZPR3d expression was observed in the young stele (below emerging lateral roots), but was not detectable in the root cap and most of the meristematic region (Figure 5B,D). GmZPR3d expression was not detected in emerging lateral roots (Figure 5F), but was clearly visible in emerging nodules, particularly in the nodule primordium region (Figure 5H). In cross sections of mature nodules, GmZPR3d expression was primarily detected in parenchyma tissues adjacent to the central zone (Figure 5I). These results indicate an overlapping expression of GmZPR3d and GmHD-ZIP III-1 and -2, notably in root vascular tissues, and the nodule parenchyma.

### 2.4. Over-Expression of GmZPR3d Alters Root Vascular Development

**Figure 6:**
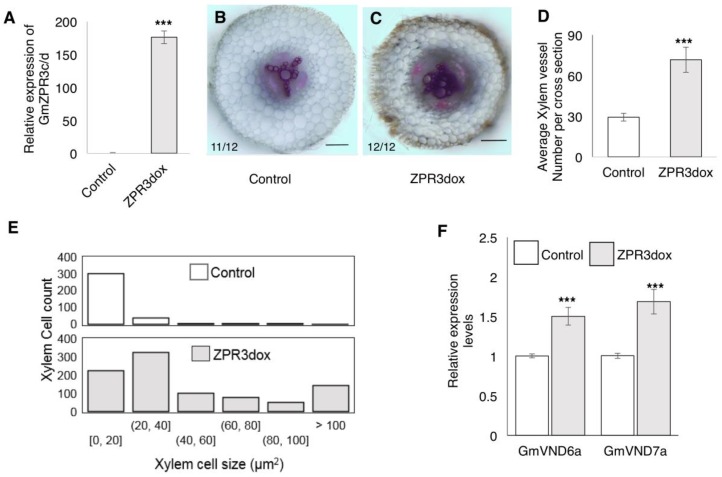
Ectopic xylem development in ZPR3d-overexpressing soybean roots. (**A**) Relative expression levels of ZPR3c/d in CsVMV:ZPR3d over expression roots (ZPR3dox) compared to vector control roots (fold change), assayed by RT-qPCR. Data shown are average of three independent biological replicates. Error bars indicate SE. *** *P* < 0.001, Student’s *t*-test. (**B**,**C**) Transverse sections of (**B**) vector control, and (**C**) ZPR3dox roots above the first emerged lateral root stained with phloroglucinol. The number of independent transgenic root sections showing the representative pattern out of the total number evaluated are indicated. Scale bars are 200 µm. (**D**) Number of xylem cells in each transverse section of vector control and ZPR3dox roots above the first emerged lateral root. Data shown are average of 12 transverse sections per construct each from an independent root (*n* = 12 roots/construct) and error bars indicate SE. *** *P* < 0.001, Student’s *t*-test. (**E**) Distribution of xylem cell sizes in transverse sections of vector control and ZPR3dox roots above the first emerged lateral root measured using imageJ. Data shown are from 351 cells for vector control and 860 cells for ZPR3dox measured in 12 transverse sections each from an independent root for each construct. (**F**) Relative expression levels of GmVND6a and GmVND7a in ZPR3dox roots compared to vector control (fold change), assayed by RT-qPCR. Data shown are average of three independent biological replicates. Error bars indicate SE. *** *P* < 0.001, Student’s *t*-test.

Overlapping expression patterns of the interacting partners GmZPR3d and GmHD-ZIP III-1 and -2 in the root stele and nodule parenchyma, suggested that GmZPR3d might regulate the activity of GmHD-ZIP III-1 and -2 in these tissues. To determine the functional role of GmZPR3d, we over-expressed this gene (ZPR3dox) in composite soybean plant roots. Over-expression was validated using RT-qPCR assays that showed a ~170-fold increase in the expression levels in ZPR3dox roots (Figure 6A). Transverse sections of ZPR3dox roots just above the first emerged lateral roots showed altered vascular development (Figure 6B vs. Figure 6C; Figure S5). In vector control roots, metaxylem cells were present in the middle and were surrounded by the protoxylem interspersed with cambial and phloem cells resulting in typical triarch (Figure 6B) or tetrarch (not shown) xylem patterning. In contrast, ZPR3dox roots displayed ectopic secondary xylem formation obscuring the typical xylem pattern (Figure 6C). Secondary xylem formation was present in younger region of the root in ZPR3dox roots vs. control roots.

We counted the number of tracheary elements (xylem cells) and measured their area in cross section images of 12 roots each for vector control and ZPR3dox. ZPR3dox roots had ~2.5-fold higher number of xylem cells (Figure 6D) compared to vector control roots. We also observed a significant increase in the average xylem cell size (cross sectional area) in ZPR3dox roots. It was evident from the pattern of xylem cell size that a large proportion of these cells in ZPR3dox roots were larger (Figure 6E). For example, ~87% of xylem cells in vector control roots were 0–20 µm^2^ in size. On the other hand, only ~26% of xylem cells in ZPR3dox roots were 0–20 µm^2^ in size with nearly 16% in the >100 µm^2^ range. The ectopic secondary xylem formation was reminiscent of what has been observed in arabidopsis plants with reduced levels of *ATHB15* [26,27,28] and miR166 over-expressing *M. truncatula* roots [18]. This indicated that the observed phenotypes are likely to be due to impaired HD-ZIP III function, in particular that of GmHD-ZIP III-2 (a potential ortholog of ATHB15) resulting from over-expression of GmZPR3d.

To evaluate if the xylem phenotypes arose from disruption of GmHD-ZIP III-2 activity, we measured the expression levels of key marker genes in xylem cell identity pathways acting downstream of HD-ZIP III genes. In Arabidopsis, VND6 and VND7 are vessel-specific master switches for metaxylem and protoxylem respectively [29,30]. Over-expression of VND6 and VDN7 in Arabidopsis resulted in transdifferentiation of various cell types into xylem cells resulting in increased xylem formation similar to that in ZPR3dox soybean roots. ATHB15 represses the master switches VND6 and VND7 and other downstream genes [31]. Therefore, impaired GmHD-ZIP III-2 activity can be expected to result in increased VND6 and VND7 expression levels. We identified the potential soybean orthologs of VND6 and VND7 and evaluated their expression in ZPR3dox roots using RT-qPCR. There was a 1.5- and 1.6-fold increase in the levels of GmVND6a and GmVND7a transcripts respectively in GmZPR3d over expression roots compared to the vector control roots (Figure 6F). This suggested that the ectopic secondary xylem formation in ZPR3dox roots is likely due to impaired GmHD-ZIP III-2 activity.

### 2.5. Over-Expression of GmZPR3d Alters Nodule Size and Vascular Bundle Branching

**Figure 7:**
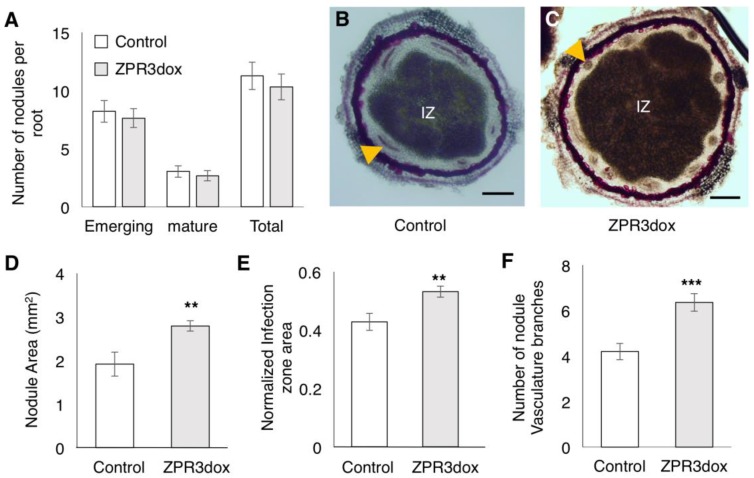
ZPR3d overexpression alters nodule size and vasculature. (**A**) Number of emerging, mature, and total nodules on Vector control and ZPR3dox roots. No significant difference observed based on Poisson distribution analysis. Data shown are average from at least 140 independent transgenic roots for each construct. Error bars indicate SE. (**B**,**C**) Cross sections of representative nodules from (**B**) vector control and (**C**) ZPR3dox roots sectioned transversally to the nodule axis and stained with phloroglucinol. A nodule vascular bundle is indicated by the yellow arrowhead; IZ – Infection Zone (central zone). Scale bars are 200µM. (**D**,**E**) Nodule area and infection zone area normalized as a proportion of nodule area in nodules from vector control and ZPR3dox roots. Data shown are averages (*n* = 10 sections per construct each from an independent nodule) and error bars indicate SE. ** *P* < 0.01, Mann-Whitney-Wilcox test. (**F**) Number of nodule vascular bundles that has branched and is visible in median transversal sections of nodules from vector control and ZPR3dox roots. Data shown are averages (*n* = 10 sections per construct each from an independent nodule) and error bars indicate SE. *** *P* < 0.001 Student’s *t*-test.

To evaluate the impact of GmZPR3d over-expression on nodule development, we inoculated composite soybean plants with *B. diazoefficiens*, and counted the number of nodules and evaluated their structure. Nodules were classified into emerging nodules which appeared as slight bump on the root surface, and mature nodules that were spherical in shape and had emerged out of the root. We did not observe any significant difference in the numbers of emerging or mature nodules between vector control and ZPR3dox roots (Figure 7A). We further evaluated the structure of mature nodules on ZPR3dox roots by measuring nodule size, central zone (infection zone) size, and counting the number of nodule vascular bundle branches in transverse sections of mature nodules stained with phloroglucinol (Figure 7B,C and Figure S6). GmZPR3d over-expression led to a significant increase in the nodule area (~1.5 fold) and normalized central zone (infection zone) area (~1.3 fold) compared to the vector control (Figure 7D,E). We also observed a significant increase in the number of vascular bundle branches in ZPR3dox nodules compared to control nodules (Figure 7F). This suggested that HD-ZIP III activity might be crucial for regulating nodule and central zone size as well as vascular bundle branching in the nodules.

### 2.6. ZPR3d Interacts with A Number of Soybean Proteins

Microproteins such as ZPR are known to influence the activity of several transcriptional factors and signaling proteins [32]. Results from single-molecule pull-down assays suggested that AtZPR3 and PHBs form a heterotetramer complex [33]. To determine other potential interacting partners of ZPR3d in soybean, we performed a global yeast two-hybrid screen. Upon screening 80.5 million interactions, 380 positive interactions were identified and the clones were sequenced (Table S2). Only two genes were present in the highest confidence interaction category: Glyma17g12110.2 (WUS-2 interacting protein) and Glyma03g28460 (SEC14 cytosolic factor family protein). We identified other interacting partners in categories B and C, many of them containing a potential leucine zipper motif or a coiled coil domain. Finally, there were 51 interacting partners in category D, which included a HD-ZIP III gene (Glyma08g21610/GmHD-ZIP III-10, a close paralog of GmHD-ZIP III-2; Table S2). This indeed suggested that GmZPR3d might regulate a number of other pathways.

Proteins with direct physical interaction or indirect functional interaction appear to have a higher likelihood of being co-expressed or co-regulated [34]. To rank ZPR3d interactors to identify potential candidates for in planta interaction assays in the future, we performed a co-expression analysis among the GmZPR3d interactors along with all soybean HD-ZIP III and ZPR genes using expression patterns observed in the soybean gene atlas [35]. We observed a high level of correlation among the interactors of GmZPR3d compared to two randomly chosen gene sets of the same size from the soybean genome (Figure S3A). This provided additional confidence that the expression of GmZPR3d and some of its interactors might be co-regulated and therefore they might act together to regulate additional pathways. When the interactors were ranked based on correlation co-efficient values vs. GmZPR3d (Figure S3B; Table S3), the two high-confidence GmZPR3d interactors ranked relatively higher and showed positive correlation (*r* = 0.28, Glyma03g28460 and *r* = 0.11, Glyma17g12110). Other potential candidates for in planta validation included another WUS-interacting protein (Glyma13g22720, *r* = 0.54), Tudor superfamily protein (Glyma20g34040, *r* = 0.54), a BCL-2 associated athanogene (Glyma18g43530, *r* = 0.54), PHD/F-box domain protein (Glyma14g11400; *r* = 0.34).

## 3. Discussion

Interaction between Arabidopsis ZPRs and HD-ZIP III proteins have been evaluated through yeast two-hybrid and in vitro pull-down assays [19,36]. AtZPR3 showed positive interaction with four of the five AtHD-ZIP III proteins (except REV) in yeast two-hybrid assays. However, positive interaction between AtZPR3 and REV was established through an in vitro pull down assay [36]. All the four classes of Arabidopsis ZPR proteins bind to all five HD-ZIP III monomers using in vitro pull down assays [19]. In this study, we identified eight soybean ZPR orthologs and evaluated their interactions with GmHD-ZIPIII-1 (a potential ortholog of PHB and PHV) and GmHD-ZIPIII-2 (potential ortholog of ATHB15) using yeast two-hybrid assays. Positive interactions were observed only for three of the eight GmZPRs. GmZPR3b, 3c and 3d showed positive interaction with both GmHD-ZIP III-1 and -2, while the other ZPRs showed no interaction. It is notable that despite identical amino acid sequence in the heptad repeats of the potential leucine-zipper domain, GmZPR3a failed to show positive interaction with GmHD-ZIP III-1 or -2 while GmZPR3b was able to interact with both these GmHD-ZIP IIIs. This suggested that amino acids outside (downstream) of the leucine heptad repeats might determine interaction potential likely due to potential structural hindrances. Similarly, none of the GmZPR1 proteins (that possessed an N-terminal domain unlike ZPR3 proteins) interacted with the two GmHD-ZIP IIIs tested. It is possible that the non-interacting ZPRs interact with a set of HD-ZIP IIIs that were not evaluated in this study or that the interactions may only be detected via in vitro pull down or in planta assays as was the case for AtZPR3 and AtREV. In addition, the strength of interaction, in particular with GmHD-ZIP III-2 appeared to be different among GmZPR3b, 3c and 3d with ZPR3d being the strongest. Differences in interaction strength have not been reported for Arabidopsis ZPR and HD-ZIP III pairs. If confirmed through in vitro pull down and/or in planta interaction assays, such differences in the ability of different ZPR proteins to interact with different HD-ZIP III proteins and differences in interaction strength would suggest an added layer of complexity in HD-ZIP III regulation. It would also be interesting to evaluate if such differences evolved in specific plant lineages (e.g., legumes) or are observed in most plant species.

Spatio temporal expression patterns of GmZPR3d and GmHD-ZIP III-1 and -2 indicated overlapping expression of these genes in the root stele (Figure 3). In agreement, we observed significant alterations in the root vascular development in ZPR3dox roots (Figure 6). Similar alterations in vascular development (ectopic secondary xylem) and additional pattering defects were observed in *M. truncatula* roots over-expressing microRNA166 [18]. The expression levels of multiple HD-ZIP III genes were reduced in these roots suggesting that the phenotype resulted from reduced HD-ZIP III activity. In Arabidopsis, ATHB8 appears to promote xylem differentiation where as ATHB15 inhibits this process [28]. Antisense-mediated suppression of ATHB15 resulted in enhanced xylem differentiation, formation of interfascicular tissue, and increased lignification in the inflorescence stem. These data suggested that the ectopic secondary xylem formation in younger regions of soybean roots over-expressing GmZPR3d might primarily be due to suppression of GmHD-ZIP III-2 activity (potential soybean ortholog of ATHB15). Additional evidence for this conclusion came from qPCR assays that showed upregulation of GmVND6 and GmVND7 (xylem-specific master switches that confer cell identity) in ZPR3dox roots.

We also observed overlapping expression of GmZPR3d and the tested HD-ZIP IIIs in the nodule parenchyma and its interface with the central zone. We observed increased nodule and central zone area as well as an increase in the number of nodule vascular bundles in mature nodules of ZPR3dox roots. Interestingly, suppression of ATHB15 also led to an increase in the number of vascular bundles in the inflorescence stems [27,28]. The authors of the arabidopsis study propose that the increase might be due to increased stem size resulting from fasciation. However, we observed no impact on nodule vascular branch numbers when nodule size was reduced due to suppression of auxin-amino acid conjugating GH3 genes [37]. It remains to be seen if the increase in the number of nodule vascular bundles is due to increased nodule size or not. Therefore, the majority of the observed phenotypes in ZPR3dox roots and nodules might be primarily due to the inhibition of GmHD-ZIP III-2 activity. Notably, we observed strongest interaction between GmZPR3d and GmHD-ZIP III-2 in yeast two-hybrid assays. GmZPR3d might be capable of interacting preferentially with GmHD-ZIP III-2 in soybean, and that this interaction plays a key role in determining root xylem differentiation, nodule and central zone (infection zone) size, and nodule vascular branching.

In the global yeast two-hybrid screen, the two high confidence interactors did not have any predicted leucine zipper domains. However, Glyma17g12110, annotated as a WUS-interacting protein had WD40-like repeats. Proteins with WD repeats do anchor protein complexes and it is possible that this ZPR3d interactor serves such a role [38]. Interaction between a HD-ZIP I protein and a WD repeat containing protein has been demonstrated in cucumber [39]. The other interactor encoded by Glyma03g28460, annotated as a SEC14 family protein has a CRAL-TRIO domain that binds small lipophilic molecules. It is interesting to note that HD-ZIP III proteins also have a lipid-binding domain. However, SEC14 family proteins are typically cytosolic while ZPR proteins are localized in the nucleus [36]. Co-expression analysis of GmZPR3d interactors also provided support for the hypothesis that these genes might be co-regulated and thus potentially act together in planta. In conclusion, our data strongly indicate a key role of GmZPR3d-GmHD-ZIP III-2 in soybean root and nodule development, and suggest a broader regulatory role for ZPR proteins in soybean.

## 4. Materials and Methods 

### 4.1. HD-ZIP III and ZPR Phylogenetic Analysis

Potential HD-ZIP III genes in soybean (*G. max*)*,* were identified and their sequences determined using peptide sequences of *A. thaliana* HD-ZIPIII genes (*PHB*, *PHV*, *REV*, *ATHB8* and *ATHB15*; www.arabidopsis.org) as query in BLASTp searches (using BLOSUM62 matrix and expected threshold value of -1) on the soybean genome ((v.189); www.phytozome.net). The sequences were evaluated for the presence of leucine zipper and homeobox domains. Peptide sequences were aligned using a gap extension penalty of 0.2 and the comparison matrix BLOSUM62 in Mega7 software [40] and a phylogenetic tree was constructed.

To identify the soybean orthologs of Arabidopsis ZPR genes BLASTp search was performed as follows. Peptide sequences of AtZPR1, AtZPR2, AtZPR3 and AtZPR4 were obtained from www.arabidopsis.org and used for BLASTp search in www.phytozome.net against *G. max* sequence using BLOSUM62 matrix and an expected threshold value of -1. Peptide alignment and phylogenetic tree construction was performed as described above for HD-ZIP IIIs. The presence of leucine residues at the heptad position shown to form the hydrophobic core of the two α-helix structure was manually evaluated.

### 4.2. Cloning and Construction of DNA Vectors

For yeast two-hybrid interaction assays, primers were designed to amplify the coding sequences of GmHD-ZIPIII-1 and -2 for cloning into pGADT7 vector/ prey vector. Similarly, primers were designed to clone all eight GmZPR genes into pGBKT7. The genes were amplified using Advantage HD polymerase (Clontech, CA, USA) and the PCR products were purified using agarose gel electrophoresis and Wizard SV gel & PCR cleanup system (Catalogue # A9282, Promega, WI, USA). The purified products were cloned in to the respective pGADT7 and pGBKT7 vectors using the gene infusion protocol (Clontech, CA, USA), and sequenced were verified.

An ~1700bp upstream regions of GmHD-ZIP III-1, -2, and GmZPR3d were amplified from soybean genomic DNA using Platinum high fidelity supermix (Cat # 10790-020, Invitrogen, CA, USA). The PCR product was cloned into pCR8/GW/TOPO-TA vector (Thermofisher Scientific, Waltham, MA, USA) and its sequence was verified. The promoter fragment was cloned into the destination vector, pCAMGFP-GW:GUS using Gateway LR clonase II enzyme mix following the manufacturer’s protocol (Thermofisher Scientific, Waltham, MA, USA) to obtain pCAMGFP-Prom:tdTomato vectors.

To over-express GmZPR3d in soybean composite plant roots, its coding sequence was amplified and cloned into pCR8/GW/TOPO-TA (Thermofisher Scientific, Waltham, MA, USA), sequence verified, and cloned into pCAMGFP-CsVMV:Gw [25] using Gateway LR clonase II enzyme mix (Thermofisher Scientific, Waltham, MA, USA) to obtain pCAMGFP-ZPR3dox binary vector. The primers used in this study are listed in Table S4.

### 4.3. One-on-One Yeast Two-Hybrid Assays

The sequence verified clones were transformed into yeast cells (Y2H gold for pGBKT7 vector with ZPR genes and Y187 for pGADT7 vector with GmHD-ZIPIII-1 and 2) using protocols described in Yeastmaker^Tm^ Yeast Transformation system 2 user manual (Clontech, CA, USA). Yeast mating and screening of the interaction partners using different selection media was performed following the user manual of Matchmaker^®^ Gold Yeast Two-Hybrid System (catalogue #630489, Clontech, CA, USA). All growth assays were reconfirmed using three independent yeast transformants. Enzymatic assays to determine the strength of interaction were performed in triplicate according to the “Yeast protocol handbook” (Clontech, CA, protocol PT4084-1, USA) and using 2-Nitrophenyl β-D-Galactopyranoside/ONPG (Catalogue# 73660, Clontech, CA, USA) as the substrate. Millers units were calculated and statistical significance was determined using one-way ANOVA and Tukey HSD in JMP software (SAS Institute Inc., Cary, NC, USA, 1989-2019).

### 4.4. Plant Materials and Composite Plant Generation

*Glycine max* cv. Williams-82, the genotype used for soybean genome sequencing project [41] was used in this study. The seeds were surface-sterilized by rinsing with 8% clorox for 4 min and 70% ethanol for 4 min followed by thorough rinsing with distilled water for 8–10 times to remove any residual bleach/ethanol. Seeds were germinated in 4” pots filled with a mixture of vermiculite: perlite (Hummert International, St. Louis, MO, USA) in the ratio of 1:3 and watered with Hoagland nutrient solution. The plants were grown in a controlled environment vertical growth chamber (Conviron Growth chamber, Winnipeg, Manitoba, Canada). Growth conditions used were: 16 h light and 8 h dark and 50% relative humidity with a day and night temperature of 25 and 20 °C respectively.

### 4.5. Plant Transformation and Nodulation Assay

*Agrobacterium rhizogenes* K599 cells were transformed with the construct of interest by electroporation [42]. Hairy root composite plant transformation was performed following the protocol described previously [43] using 12–14 days old soybean seedlings as explants. Twenty-one days after transformation, the plants produced adventitious roots including *A. rhizogenes*-induced transgenic roots. GFP positive roots carrying the transgene of interest were selected by screening for epifluorescence using the FITC filter in an SZX16 stereo microscope (Olympus Corporation, Shinjuku, Tokyo, Japan).

For nodulation assays, the screened plants were transferred to 4” pots filled with sterilized 3:1 vermiculite: perlite mix. Five days post transfer, the plants were inoculated with *Bradyrhizobium diazoefficiens USDA110* cells re-suspended in nitrogen free plant nutrient solution (N^−^ PNS) to an OD_600nm_ of 0.08 [25,44]. About 25 mL of this suspension was added uniformly to each pot. For mock-inoculated plants, the same quantity of N^−^ PNS was applied. Transgenic roots were harvested under an epifluorescence microscope at 14–17 dpi and the nodules were counted. Nodules were classified as “emerging” if they appeared as a bump on root surface and “mature” if they were completely protruded out of the root surface. At least 140 independent transgenic roots were evaluated for each construct. The statistical significance of difference in nodule numbers if any between over expression roots and vector control roots was determined using zero inflated Poisson distribution package available in R statistical software.

### 4.6. Staining, Microscopy and Image Analysis

To evaluate xylem structure, roots were hand-sectioned transversally just above the youngest lateral root, and stained with a saturated solution of phloroglucinol prepared in 20% HCl. The stained sections were mounted immediately 20% HCl and imaged within 10 min of staining to avoid tissue damage. All the images were taken with a resolution of 4080 × 3072 pixels using an AX70 upright microscope, DP70 digital camera and cellSens software (Version 2.1, Olympus, Shinjuku, Tokyo, Japan), and saved as tiff files. One section each from 12 independent transgenic roots were analyzed for each construct from at least 7 plants. No alterations or image enhancements were performed on the images before analysis. In each cross section imaged, xylem cell boundaries were manually drawn using ImageJ, and the same software was used to determine the xylem cell area and also to count the number of xylem cells [45]. Statistical analysis was performed in Microsoft excel Student’s *t*-test or using R version 3.0 for the Shapiro-Wilk or Mann-Whitney-Wilcoxon tests.

To assess nodule structure, mature nodules were hand-sectioned transversally to the nodule axis, and the sections were stained with a saturated solution of phloroglucinol prepared in 20% HCl. The stained sections were immediately mounted and imaged as described above for root sections. One section each from 10 independent transgenic nodules obtained from at least 7 different plants were analyzed for each construct. No alterations or image enhancements were performed on the images before analysis. The number of nodule vasculature branches were counted manually using the microscopic images. The cross-section area of the nodule and central infection zone were measured using imageJ software. For statistical analysis, normality of data distribution was first evaluated using Shapario-wilk test in *R* (version 3.0, R-Foundation for statistical computing, Vienna, Austria). Statistical significance of differences was evaluated using Student’s *t*-test in Microsoft excel (normally distributed data) or Mann-Whitney-Wilcox test in *R* (not normally distributed).

For epifluorescence microscopy to visualize promoter:tdTomato expression patterns, whole roots or root segments were mounted in 10% glycerol and imaged using AX70 upright microscope (Olympus, Shinjuku, Tokyo, Japan) using the TRITC/PI filter (exposure time: 25 ms). Images were taken at a resolution of 2040 × 1536 pixels and saved as tiff files. Mature nodules were sectioned transversally, sections were mounted in 10% glycerol, and imaged in an Olympus FV1200 scanning confocal microscope (Olympus, Shinjuku, Tokyo, Japan). The 488 nm Ar laser (8% power) and default settings for GFP in FV10-ASW software were used for GFP imaging. The 543 nm HeNe laser (8% power) and default settings for DsRed in FV10-ASW software were used for tdTomato imaging, Images were taken at a resolution of 663 × 663 pixels, and a maximum intensity projection stack of 5 slices (5 µm total depth) were exported as a tiff file for each nodule section.

### 4.7. Gene Expression Analysis by RT-qPCR

ZPR3dox and vector control (pCAMGFP-CsVMV:GUS), plants were generated using hairy root transformation and whole transgenic roots were harvested from uninoculated roots. The root tissues were immediately frozen in liquid nitrogen, and stored at −80 °C until further analysis. RNA isolation, reverse transcription, and qPCR assays for gene expression were performed as previously described [25]. Gene expression levels were normalized to those of housekeeping genes GmACTIN, GmCONS7, and GmCONS15 [46]. Data shown are relative expression levels to that of GmACTIN, averaged from three biological replicates with three technical replicates each. Statistical significance of differences in gene expression was evaluated using Student’s *t*-test, after verifying the data distribution using Shapiro-Wilk test in *R* software version 3.0. qPCR primers used in this study are listed in Table S4.

### 4.8. Genome-Wide Yeast Two-Hybrid Assay

Full length coding sequence of GmZPR3d was cloned into pB27 (N-LexA-ZPR3d-C fusion) and used as the bait. The prey libraries were prepared in pB27A (Gal4-AD fusions) using shoot and root tissues of mock- and *B. diazoefficiens*-inoculated (harvested at 14 dpi) and uninoculated soybean seedlings (cv. Williams 82). Interaction assay was performed by Hybrigenics services (Hybrigenics, France) evaluating for successful activation of *HIS3* and *LacZ* (https://www.hybrigenics-services.com/contents/resources/yeast-two-hybrid-principle). The interacting partners were classified based on the confidence of interaction into four different classes (A–D) based on the number different library clones that showed positive interaction with A indicating high confidence, and D with low confidence [47]. Sequences from positive clones were obtained and the Gene ID and annotation were predicted using BLAST search against soybean coding sequence (v.189) in www.phytozome.net. For identifying if the coding sequence containing any leucine zipper or coiled coil domains, the sequences were analyzed using 2ZIP tool (http://2zip.molgen.mpg.de/).

## Supplementary Materials

Supplementary materials can be found at http://www.mdpi.com/1422-0067/20/4/827/s1.

## Abbreviations

ZPR	Small Leucine Zipper
HD-ZIP III	Class III Homeodomain Leucine Zipper
BLAST	Basic Local Alignment Search Tool
SD	Synthetic Dropout
Leu	Leucine
Trp	Tryptophan
QDO	Quadruple Drop Out
AbA	Aureobasidin
GFP	Green Fluorescent Protein

## References

[1] Hawker N.P., Bowman J.L. (2004). Roles for Class III HD-ZIP and KANADI Genes in arabidopsis Root Development. Plant Physiol..

[2] Izhaki A., Bowman J.L. (2007). KANADI and Class III HD-ZIP Gene Families Regulate Embryo Patterning and Modulate Auxin Flow during Embryogenesis in arabidopsis. Plant Cell.

[3] Emery J.F., Floyd S.K., Alvarez J., Eshed Y., Hawker N.P., Izhaki A., Baum S.F., Bowman J.L. (2003). Radial Patterning of Arabidopsis Shoots by Class III HD-ZIP and KANADI Genes. Curr. Biol..

[4] Elhiti M., Stasolla C. (2009). Structure and function of homodomain-leucine zipper (HD-ZIP) proteins. Plant Signal. Behav..

[5] Ariel F.D., Manavella P.A., Dezar C.A., Chan R.L. (2007). The true story of the HD-ZIP family. Trends Plant Sci..

[6] Magnani E., Barton M.K. (2011). A Per-ARNT-Sim-Like Sensor Domain Uniquely Regulates the Activity of the Homeodomain Leucine Zipper Transcription Factor REVOLUTA in arabidopsis. Plant Cell Online.

[7] Prigge M.J., Otsuga D., Alonso J.M., Ecker J.R., Drews G.N., Clark S.E. (2005). Class III Homeodomain-Leucine Zipper Gene Family Members Have Overlapping, Antagonistic, and Distinct Roles in arabidopsis Development. Plant Cell Online.

[8] Baima S., Possenti M., Matteucci A., Wisman E., Altamura M.M., Ruberti I., Morelli G. (2001). The arabidopsis ATHB-8 HD-Zip Protein Acts as a Differentiation-Promoting Transcription Factor of the Vascular Meristems. Plant Physiol..

[9] Ferguson B.J., Indrasumunar A., Hayashi S., Lin M.H., Lin Y.H., Reid D.E., Gresshoff P.M. (2010). Molecular analysis of legume nodule development and autoregulation. J. Integr. Plant Biol..

[10] Hirsch A.M. (1992). Developmental biology of legume nodulation. New Phytol..

[11] Brown S., Walsh K. (1994). Anatomy of the Legume Nodule Cortex With Respect to Nodule Permeability. Funct. Plant Biol..

[12] Walsh K.B., Atkins R.S., Low C.S. (1992). Vascular anatomy of fabaceous nodules of determinate growth. Plant Cell Environ..

[13] Frugier F., Kosuta S., Murray J.D., Crespi M., Szczyglowski K. (2008). Cytokinin: secret agent of symbiosis. Trends Plant Sci..

[14] Suzaki T., Ito M., Kawaguchi M. (2013). Genetic basis of cytokinin and auxin functions during root nodule development. Front. Plant Sci..

[15] Heckmann A.B., Lombardo F., Miwa H., Perry J.A., Bunnewell S., Parniske M., Wang T.L., Downie J.A. (2006). Lotus japonicus nodulation requires two GRAS domain regulators, one of which is functionally conserved in a non-legume. Plant Physiol..

[16] Nontachaiyapoom S., Scott P.T., Men A.E., Kinkema M., Schenk P.M., Gresshoff P.M. (2007). Promoters of orthologous Glycine max and Lotus japonicus nodulation autoregulation genes interchangeably drive phloem-specific expression in transgenic plants. Mol. Plant Microbe Interact..

[17] Mao G., Turner M., Yu O., Subramanian S. (2013). miR393 and miR164 influence indeterminate but not determinate nodule development. Plant Signal. Behav..

[18] Boualem A., Laporte P., Jovanovic M., Laffont C., Plet J., Combier J.P., Niebel A., Crespi M., Frugier F. (2008). MicroRNA166 controls root and nodule development in Medicago truncatula. Plant J..

[19] Wenkel S., Emery J., Hou B.-H., Evans M.M.S., Barton M.K. (2007). A Feedback Regulatory Module Formed by LITTLE ZIPPER and HD-ZIPIII Genes. Plant Cell Online.

[20] Brandt R., Salla-Martret M., Bou-Torrent J., Musielak T., Stahl M., Lanz C., Ott F., Schmid M., Greb T., Schwarz M. (2012). Genome-wide binding-site analysis of REVOLUTA reveals a link between leaf patterning and light-mediated growth responses. Plant J..

[21] Deppmann C.D., Acharya A., Rishi V., Wobbes B., Smeekens S., Taparowsky E.J., Vinson C. (2004). Dimerization specificity of all 67 B-ZIP motifs in Arabidopsis thaliana: A comparison to Homo sapiens B-ZIP motifs. Nucleic Acids Res..

[22] Landschulz W.H., Johnson P.F., McKnight S.L. (1988). The leucine zipper: a hypothetical structure common to a new class of DNA binding proteins. Science.

[23] Damodaran S., Subramanian S. (2016). South Dakota State University, Brookings, SD. Unpublished observations from yeast-2-hybrid assays between GmZPR and GmHD-ZIP III proteins.

[24] Miller J.H. (1972). Experiments in Molecular Genetics.

[25] Turner M., Nizampatnam N.R., Baron M., Coppin S., Damodaran S., Adhikari S., Arunachalam S.P., Yu O., Subramanian S. (2013). Ectopic expression of miR160 results in auxin hypersensitivity, cytokinin hyposensitivity, and inhibition of symbiotic nodule development in soybean. Plant Physiol..

[26] Ramachandran P., Carlsbecker A., Etchells J.P. (2017). Class III HD-ZIPs govern vascular cell fate: an HD view on patterning and differentiation. J. Exp. Bot..

[27] Du Q., Avci U., Li S., Gallego-Giraldo L., Pattathil S., Qi L., Hahn M.G., Wang H. (2015). Activation of miR165b represses AtHB15 expression and induces pith secondary wall development in Arabidopsis. Plant J..

[28] Kim J., Jung J.-H., Reyes J.L., Kim Y.-S., Kim S.-Y., Chung K.-S., Kim J.A., Lee M., Lee Y., Kim V.N. (2005). microRNA-directed cleavage of ATHB15 mRNA regulates vascular development in Arabidopsis inflorescence stems. Plant J..

[29] Kubo M., Udagawa M., Nishikubo N., Horiguchi G., Yamaguchi M., Ito J., Mimura T., Fukuda H., Demura T. (2005). Transcription switches for protoxylem and metaxylem vessel formation. Genes Dev..

[30] Yamaguchi M., Goue N., Igarashi H., Ohtani M., Nakano Y., Mortimer J.C., Nishikubo N., Kubo M., Katayama Y., Kakegawa K. (2010). VASCULAR-RELATED NAC-DOMAIN6 and VASCULAR-RELATED NAC-DOMAIN7 effectively induce transdifferentiation into xylem vessel elements under control of an induction system. Plant Physiol..

[31] Du Q., Wang H. (2015). The role of HD-ZIP III transcription factors and miR165/166 in vascular development and secondary cell wall formation. Plant Signal. Behav..

[32] Staudt A.-C., Wenkel S. (2011). Regulation of protein function by “microProteins”. EMBO Rep..

[33] Husbands A.Y., Aggarwal V., Ha T., Timmermans M.C.P. (2016). In Planta Single-Molecule Pull-Down Reveals Tetrameric Stoichiometry of HD-ZIPIII:LITTLE ZIPPER Complexes. Plant Cell.

[34] Xulvi-Brunet R., Li H. (2010). Co-expression networks: Graph properties and topological comparisons. Bioinformatics.

[35] Libault M., Farmer A., Joshi T., Takahashi K., Langley R.J., Franklin L.D., He J., Xu D., May G., Stacey G. (2010). An integrated transcriptome atlas of the crop model Glycine max, and its use in comparative analyses in plants. Plant J..

[36] Kim Y.-S., Kim S.-G., Lee M., Lee I., Park H.-Y., Seo P.J., Jung J.-H., Kwon E.-J., Suh S.W., Paek K.-H. (2008). HD-ZIP III Activity Is Modulated by Competitive Inhibitors via a Feedback Loop in Arabidopsis Shoot Apical Meristem Development. Plant Cell Online.

[37] Damodaran S., Westfall C., Kisely B., Jez J., Subramanian S. (2017). Nodule-Enriched GRETCHEN HAGEN 3 Enzymes Have Distinct Substrate Specificities and Are Important for Proper Soybean Nodule Development. Int. J. Mol. Sci..

[38] Van Nocker S., Ludwig P. (2003). The WD-repeat protein superfamily in Arabidopsis: conservation and divergence in structure and function. BMC Genomics.

[39] Chen C., Yin S., Liu X., Liu B., Yang S., Xue S., Cai Y., Black K., Liu H., Dong M. (2016). The WD-Repeat Protein CsTTG1 Regulates Fruit Wart Formation through Interaction with the Homeodomain-Leucine Zipper I Protein Mict. Plant Physiol..

[40] Kumar S., Stecher G., Tamura K. (2016). MEGA7: Molecular Evolutionary Genetics Analysis Version 7.0 for Bigger Datasets. Mol. Biol. Evol..

[41] Schmutz J., Cannon S.B., Schlueter J., Ma J., Mitros T., Nelson W., Hyten D.L., Song Q., Thelen J.J., Cheng J. (2010). Genome sequence of the palaeopolyploid soybean. Nature.

[42] Nizampatnam N.R., Schreier S.J., Damodaran S., Adhikari S., Subramanian S. (2015). microRNA160 dictates stage-specific auxin and cytokinin sensitivities and directs soybean nodule development. Plant J..

[43] Collier R., Fuchs B., Walter N., Kevin Lutke W., Taylor C.G. (2005). Ex vitro composite plants: an inexpensive, rapid method for root biology. Plant J..

[44] Bhuvaneswari T.V., Turgeon B.G., Bauer W.D. (1980). Early Events in the Infection of Soybean (Glycine max L. Merr) by Rhizobium japonicum: I. LOCALIZATION OF INFECTIBLE ROOT CELLS. Plant Physiol..

[45] Schneider C.A., Rasband W.S., Eliceiri K.W. (2012). NIH Image to ImageJ: 25 years of image analysis. Nat. Methods.

[46] Libault M., Thibivilliers S., Bilgin D.D., Radwan O., Benitez M., Clough S.J., Stacey G. (2008). Identification of Four Soybean Reference Genes for Gene Expression Normalization. Plant Genome.

[47] Formstecher E., Aresta S., Collura V., Hamburger A., Meil A., Trehin A., Reverdy C., Betin V., Maire S., Brun C. (2005). Protein interaction mapping: A Drosophila case study. Genome Res..

