# Supplementary material for: GmZPR3d Interacts with GmHD-ZIP III Proteins and Regulates Soybean Root and Nodule Vascular Development
Source: Int J Mol Sci. 2019 Feb 14;20(4):827. doi: 10.3390/ijms20040827 (PMC6412583; doi:10.3390/ijms20040827)
Supplement: Supplementary file 1 [file ijms-20-00827-s001.zip › Supplementary Figures_damodaran.pdf]

Supplementary Material

GmZPR3d interacts with GmHD-ZIP III proteins and regulates soybean root and nodule vascular development

Suresh Damodaran, Amélie Dubois, Juan Xie, Qin Ma, Valérie Hindié, Senthil Subramanian

Correspondence: Senthil Subramanian: Senthil.Subramanianemail@sdstate.edu

1. Supplementary Tables

For supplementary tables, S1-S4, see attached excel workbook.

2. Supplementary Figures

A

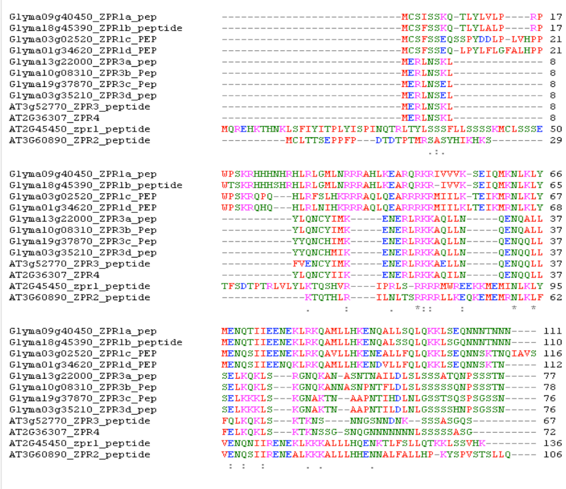

B

	GmZPR1a	GmZPR1b	GmZPR1c	GmZPR1d	GmZPR3a	GmZPR3b	GmZPR3c	GmZPR3d
GmZPR1a		93.7	63.3	65.5	23.9	23	22.6	22.6
GmZPR1b			62.2	63.8	23.3	22.4	22	22
GmZPR1c				81.4	23	20.6	21.6	21.6
GmZPR1d					19.6	20.5	19.5	22.6
GmZPR3a						91	74	72.7
GmZPR3b							76.9	76.9
GmZPR3c								92.1
GmZPR3d								

C

	abcdefg	abcdefg	abcdefg	abcdefg	abcdefg	abcdefg
GmZPR1a	KSEIQMK	NLKLYME	NQTIIEE	NEKLRKQ	AMLLHKE	NQALLSQ
GmZPR1b	KSEIQMK	NLKLYME	NQTIIEE	NEKLRKQ	AMLLHKE	NQALSSQ
GmZPR1c	KTEIKMR	NLKLYME	NQSIIEE	NEKLRKQ	AVLLHKE	NEALLFQ
GmZPR1d	LTEIKMR	NLKLYME	NQSIIEE	NQKLRKQ	AMLLHKE	NDVLLFQ
GmZPR3a	MERL	NSKLYLQ	NCYIMKE	NERLRKK	AQLLNQE	NQALLSE
GmZPR3b	MERL	NSKLYLQ	NCYIMKE	NERLRKK	AQLLNQE	NQALLSE
GmZPR3c	MERL	NSELYYQ	NCHIMKE	NERLRKK	AQLLNQE	NQQLLSE
GmZPR3d	MERL	NSELYYQ	NCHMIKE	NERLRKK	AQLLNQE	NQQLLSE
AtZPR3	MERL	NSKLFVE	NCYIMKE	NERLRKK	AELLNQE	NQQLLFQ
AtZPR4	MERL	NSKLYLQ	NCYIIKE	NERLRKK	AQILNQE	NQQLLFE
AtZPR1	EKKMEMI	NLKLYVE	NQNIIRE	NEKLKKK	ALLLHQE	NKTLFSL
AtZPR2	QKEMEMR	NLKLFVE	NQSIIRE	NEALKKK	ALLLHHE	NNALFAL
GmHD-ZIPIII-1	ASRLQTV	NRKLSSM	NKLLMEE	NDRLQKQ	VSQLVYD	NGFMKQQ
GmHD-ZIPIII-2	REKQRKE	SSRLQAV	NRKLTAM	NKLLMEE	NDRLQKQ	VSQLVYE
PHV	AARLQTV	NRKLNAM	NKLLMEE	NDRLQKQ	VSNLVYE	NGFMKHR

Supplementary Figure S1. Comparison of GmZPR and AtZPR peptide sequences. (A) Multiple alignment of GmZPR and AtZPR peptide sequences using CLUSTALW2 tool. B) Sequence identity matrix of GmZPR peptides. C) Prediction of Leucine zipper domains in GmZPR and GmHD-ZIPIII-1 and -2 peptides based on leucine/iso-leucine residues at position ‘d’ of the heptad series, and comparison to those of AtZPRs and PHV.

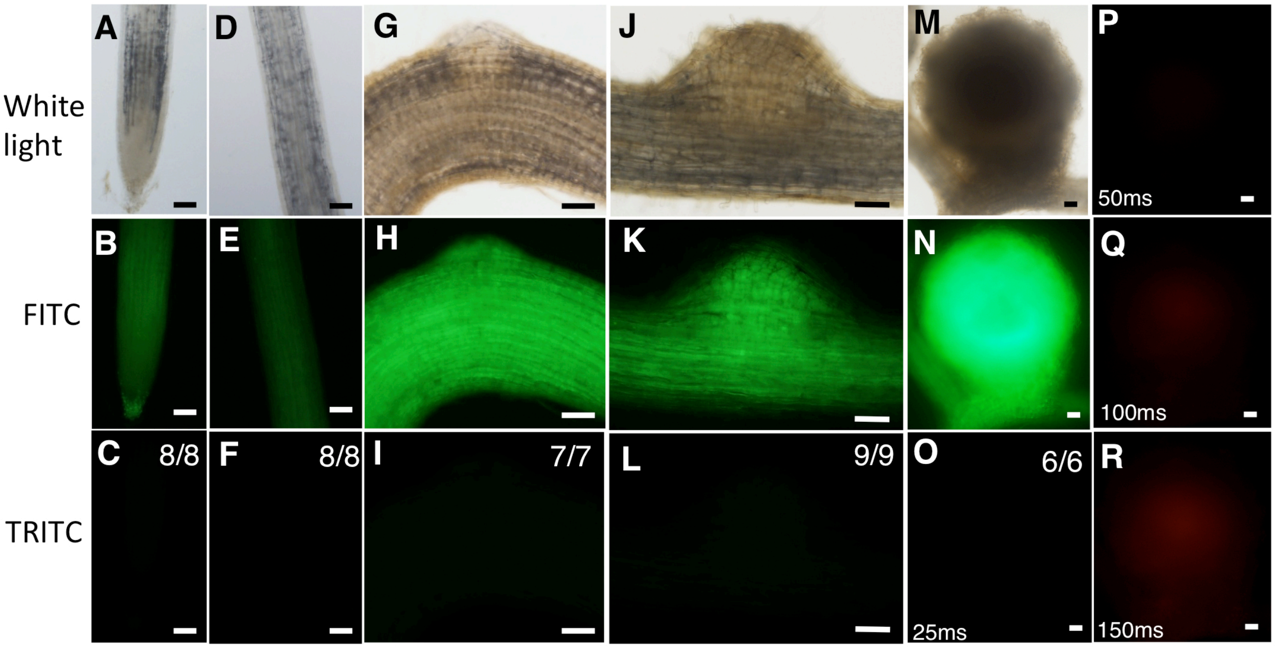

Supplementary Figure S2. Evaluation for autofluoresence in vector control roots using the TRITC Filter. Whole mounts of soybean roots and nodules expressing GFP but not tdTomato. Bright field and fluorescent images of a root tip (A, B and C), mature root region showing the root vasculature (D, E and F), an emerging lateral root (G, H and I), an emerging nodule (J, K and L), and a mature nodule (M-R) are shown. A, D, G, J and M are bright field images; B, E, H, K and N are corresponding fluorescence images obtained using a FITC filter showing GFP expression; and C, F, I, L, and O, are corresponding fluorescent images obtained using TRITC filter. Fluoresecent images in B, C, E, F, H, I, K, L, N, and O were obtained using the same imaging parameters and exposure time (25ms) used to obtain whole mount fluorescent images in Figures 3, 4, and 5. Fluorescent images in P, Q, and R correspond to M and were obtained using the TRITC filter using exposure times indicated on each panel. The number of independent transgenic roots/nodules showing the representative patterns out of the total number evaluated are indicated in panels C, F, I and L. Scale bars are 100μm (A-L) and 200μm (M-R).

.

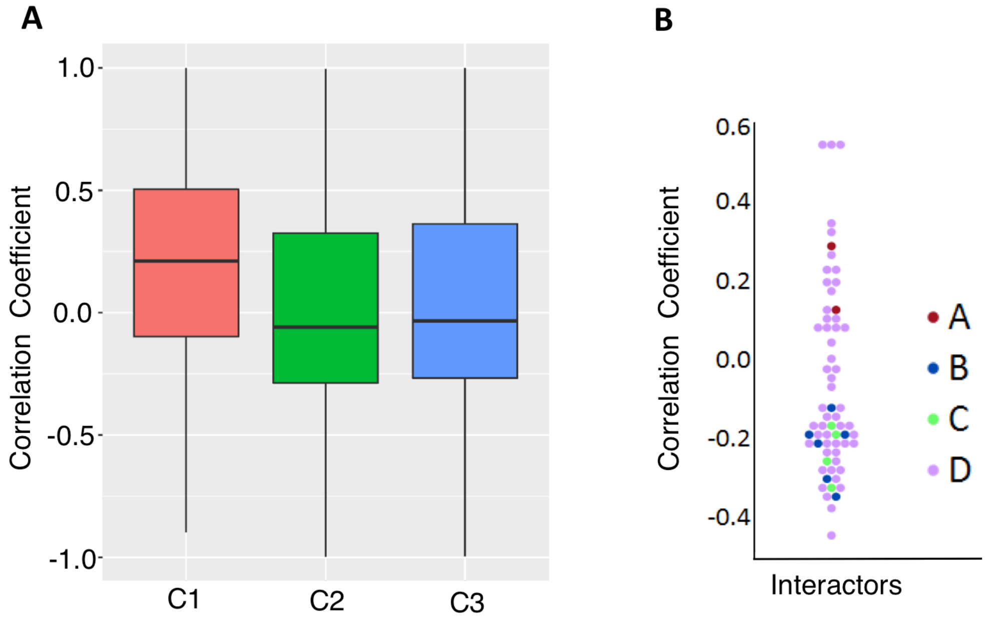

Supplementary Figure S3. Correlation of gene expression patterns among ZPR3d interactors. A) Box plots showing distribution of pair-wise Pearson correlation co-efficient scores between expression patterns of GmZPR3d interactors. Gene expression data were obtained from the soybean transcriptome atlas (Libault et al., 2010). The gene set including GmZPR3d interactors, GmZPRs, and GmHD-ZIP IIIs was denoted as X, and all the genes in the soybean genome were denoted as Y. First, pair-wise Pearson Correlation Coefficient (PCC) scores were calculated among genes in X and the generated scores were denoted as C_1. Then, |X| genes were randomly picked from Y/X (genes belong to Y but not X) and denoted as Z; Pair-wise PCC scores were calculated between the genes in X and Z (one from X and the other one from Z for each gene pair). These scores were denoted as C_2. Finally, pair-wise PCC scores were calculated among randomly selected |X| genes from Y and the scores were denoted as C_3. Pairwise Wilcoxon test indicated that C_1 vs C_2, C_2 vs C_3, and C_1 vs C_3 are significantly different (Adjusted P-value for C_1 vs C_2 and C_1 vs C_3 is $<2x10^{-16}$, and C_2 vs C_3 = 0.00945). (B) A dot plot showing Pearson correlation co-efficient scores between GmZPR3d and each of its interactors identified from the global yeast 2-hybrid screen using the gene expression data from Libault et al., 2010. The different classes of interactors based on confidence scores of global interactors are color-coded. A - Very high confidence (Red), B - high confidence (Blue), C - good confidence (Green), D - Moderate confidence (Purple).
